# Development of a Model to Estimate the Association Between Delay in Cancer Treatment and Local Tumor Control and Risk of Metastases

**DOI:** 10.1001/jamanetworkopen.2020.34065

**Published:** 2021-01-27

**Authors:** John Ng, Yael R. Stovezky, David J. Brenner, Silvia C. Formenti, Igor Shuryak

**Affiliations:** 1 Department of Radiation Oncology, Weill Cornell Medicine, New York, New York; 2 Weill Cornell Medical College, New York, New York; 3 Center for Radiological Research, Columbia University Irving Medical Center, New York, New York

## Abstract

**Importance:**

The coronavirus disease 2019 (COVID-19) pandemic has led to treatment delays for many patients with cancer. While published guidelines provide suggestions on which cases are appropriate for treatment delay, there are no good quantitative estimates on the association of delays with tumor control or risk of new metastases.

**Objectives:**

To develop a simplified mathematical model of tumor growth, control, and new metastases for cancers with varying doubling times and metastatic potential and to estimate tumor control probability (TCP) and metastases risk as a function of treatment delay interval.

**Design, Setting, and Participants:**

This decision analytical model describes a quantitative model for 3 tumors (ie, head and neck, colorectal, and non–small cell lung cancers). Using accepted ranges of tumor doubling times and metastatic development from the clinical literature from 2001 to 2020, estimates of tumor growth, TCP, and new metastases were analyzed for various treatment delay intervals.

**Main Outcomes and Measures:**

Risk estimates for potential decreases in local TCP and increases in new metastases with each interval of treatment delay.

**Results:**

For fast-growing head and neck tumors with a 2-month treatment delay, there was an estimated 4.8% (95% CI, 3.4%-6.4%) increase in local tumor control risk and a 0.49% (0.47%-0.51%) increase in new distal metastases risk. A 6-month delay was associated with an estimated 21.3% (13.4-30.4) increase in local tumor control risk and a 6.0% (5.2-6.8) increase in distal metastases risk. For intermediate-growing colorectal tumors, there was a 2.1% (0.7%-3.5%) increase in local tumor control risk and a 2.7% (2.6%-2.8%) increase in distal metastases risk at 2 months and a 7.6% (2.2%-14.2%) increase in local tumor control risk and a 24.7% (21.9%-27.8%) increase in distal metastases risk at 6 months. For slower-growing lung tumors, there was a 1.2% (0.0%-2.8%) increase in local tumor control risk and a 0.19% (0.18%-0.20%) increase in distal metastases risk at 2 months, and a 4.3% (0.0%-10.6%) increase in local tumor control risk and a 1.9% (1.6%-2.2%) increase in distal metastases risk at 6 months.

**Conclusions and Relevance:**

This study proposed a model to quantify the association of treatment delays with local tumor control and risk of new metastases. The detrimental associations were greatest for tumors with faster rates of proliferation and metastasis. The associations were smaller, but still substantial, for slower-growing tumors.

## Key Points

**Question:**

What are the potential risks of decreased local tumor control and increased metastatic spread for patients with cancer who experience treatment delays due to the coronavirus disease 2019 (COVID-19) pandemic?

**Findings:**

In this decision analytical model, a simplified mathematical model of tumor growth, metastasis formation, and tumor control by radiotherapy was developed and applied to 3 cancers with different doubling times and propensities to metastasize. Estimated detrimental risks were largest for fast-growing and intermediate-growing tumors and for longer treatment delays.

**Meaning:**

This model provided quantitative risk estimates that may help to guide physicians and patients with treatment decision-making during the COVID-19 pandemic.

## Teaser

This decision analytical model presents a simplified mathematical model of tumor growth, control, and new metastases for cancers with varying doubling times and metastatic potential and estimates tumor control probability and metastases risk as a function of treatment delay interval.

## Introduction

The effects of the coronavirus disease 2019 (COVID-19) pandemic have altered medical care globally. From March 2020 to the present, hospital systems across the United States and globally have altered their cancer care practices and functions to provide the necessary resources to handle the large number of patients with COVID-19.^1,2^ Routine procedures, including cancer screening, diagnosis, and treatments (ie, surgery, chemotherapy, and radiotherapy), have been postponed or delayed to accommodate the current situation.^3,4,5,6^ In addition, even after the peak of the pandemic, patients have continued to avoid or postpone medical center visits and treatments because of concerns about the risk of COVID-19 exposure.^4^ In parallel, sharp increases in the unemployment rate nationally will likely lead to a significant loss in access to medical care.^7,8^ It is not clear when established standards of cancer care will routinely resume, and the effects of these delays on patients with cancer remain understudied.

In response to the rapidly changing medical landscape, guidelines have been published and distributed to help oncologists determine which patients are appropriate for treatment delays.^2,6^ These global measures to contain the spread of COVID-19 have in turn focused recent attention on measuring the indirect effect of the pandemic on cancer treatment.^1,3,9,10,11,12^ In particular, there have been several recent modeling studies and expert commentaries reporting on the potential impact of cancer treatment delays due to the COVID-19 pandemic and associated mortality risks.^1,2,3,4,5,8,9,10,11,12,13^ There is general consensus on the critical importance of more high-quality data and quantitative analysis on the potential impact of treatment delays on cancer outcomes.

We propose a mathematical model, built on previously published tumor growth and control models and informed by published literature on the association of delayed treatment with cancer upstaging, to determine the potential association of cancer treatment delays with local tumor control probability (TCP) and the formation of new metastases.^14,15^ Such estimated risks may help to shape guidelines on selecting which patients should be treated without delay and those who could more safely chose to postpone treatments if necessary. We modeled 3 examples of malignant neoplasms that can grow rapidly, for which there were quality published data on tumor kinetics and for which timing of treatment initiation likely determines survival, ie, head and neck cancers, colorectal cancers, and non–small cell lung cancers.^9^

## Methods

This study was deemed exempt from institutional review board approval and the requirement for informed consent by Weill Cornell Medicine. This study followed the Consolidated Health Economic Evaluation Reporting Standards (CHEERS) reporting guideline.

### Mathematical Model of Tumor Growth and Metastasis

To illustrate the proof of principle of how substantial delays in cancer treatment could affect a patient’s risk of local tumor progression and development of new metastases, we developed a simple mathematical model of tumor growth and metastasis. The model was calibrated using data from the literature published from 2001 to 2020 and applied to an example of a fast-growing tumor (head and neck), an intermediate-growing tumor (colorectal), and a slow-growing tumor (non–small cell lung). The literature suggests that the probability of developing new metastases after delayed treatment was higher in head and neck malignant neoplasms than in lung malignant neoplasms.^16,17^ The propensity to metastasize appeared to be even higher for colorectal tumors, although there is some uncertainty behind these estimates.^18,19^

The model assumed that (1) the number of primary tumor cells as function of treatment delay time since diagnosis, *P*(*t*), grows exponentially and (2) the hazard function for metastasis formation at any given time, *M*(*t*), is proportional to the number of primary tumor cells at that time. These assumptions have clear limitations, but they provide a simplified approximation for the processes of interest with a minimal number of adjustable parameters. They are represented by the following system of differential equations, where *g* is the primary tumor growth rate and *k* is the proportionality constant for metastasis formation. Equation 1 is as follows:
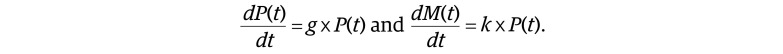
This system of equations has the following solution (Equation 2), where *P*_0_ is the initial number of primary tumor cells at *t* = 0 (ie, at cancer diagnosis) and *M_0_* is the initial metastasis hazard at *t* = 0:
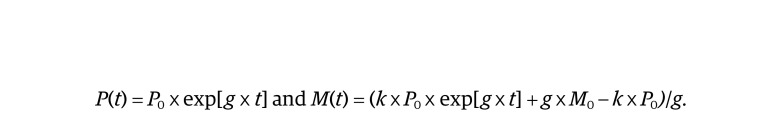
The probability of developing a new metastasis by time *t*, *PM*(*t*), can be calculated based on the hazard function *M*(*t*) in Equation 3, as follows:
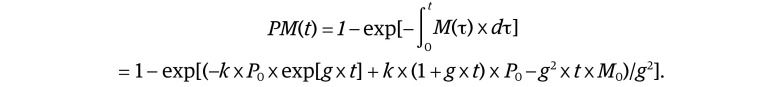
The local TCP for a tumor treated at time *t* is described by the Equation 4, based on an assumed Poisson distribution of tumor cells that survive treatment, where *S* is their surviving fraction:
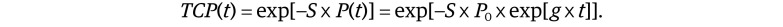
Equations 1 to 4 assume that all tumors grow at the same rate *g*. To improve the model’s realism, we added an assumption that the tumor growth rate is a normally distributed random variable with mean *G* and standard deviation σ. Then the probability of any given tumor having a growth rate *g* is *p*(*g*), described in Equation 5 as follows:
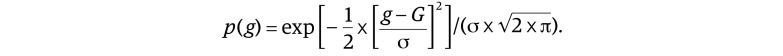
This approach accounts in a simplified way for observations that some patients have aggressive fast-growing tumors (*g* > *G*), and others have dormant ones (*g* = 0) or even spontaneous tumor regression (*g* < 0). It allows the probability distributions of the variables of interest, such as primary tumor size (*P_dist_*), metastasis probability (*PM_dist_*), and TCP (*TCP_dist_*), to be calculated as function of time (Equation 6):


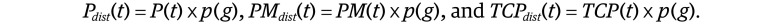


### Statistical Analysis

The model contains 5 adjustable parameters: *P*_0_, *S*, *G*, σ, and *k*. The initial number of primary tumor cells *P*_0_ was estimated based on the following assumptions: (1) the tumor cell density is 10^8^ cells/cm^−3^ for all studied cancers; (2) tumors are spherical in geometry; and (3) the initial diameter for studied lung tumors is 0.5 cm (stage I); for head and neck tumors, 3.0 cm (stage II); and for colon tumors, 5.0 cm (stage II). These values were chosen based on availability of published tumor growth rate and metastasis formation data from published peer-reviewed literature from 2001 to 2020.^16,17,18,19,20,21^ Consequently, *P*_0_ was set to 6.54 × 10^6^ cells for lung tumors, 1.41 × 10^9^ cells for head and neck tumors, and 6.54 × 10^9^ cells for colon tumors. The initial metastasis probability *M*_0_ was assumed to be 0 for each cancer type, representing the situation with no metastases at primary tumor diagnosis.

The fraction of tumor cells *S* that survive treatment was estimated for each cancer using Equation 4, substituting 0 for *t*, 0.9 for TCP (which represents the clinically plausible scenario of 90% local tumor control over 5 years for tumors treated without delay), and the cancer-specific *P*_0_ value, and solving for *S*. In other words, *S* was estimated based on a TCP of 90% at cancer diagnosis and on the previously estimated initial tumor size. *S* was 1.61 × 10^−8^ for lung cancer, 7.45 × 10^−11^ for head and neck cancer, and 1.61 × 10^−11^ for colon cancer.

The average tumor growth rate *G* and its standard deviation σ were estimated from the literature on tumor doubling times. The metastasis formation parameter *k* was estimated for each cancer based on a literature-reported metastasis probability at a specified time, given the other already described parameters.^16,17,18,19,20^

All risk estimates were calculated with normal 95% CIs. There were no inferential statistical tests used because this was not an inferential data analysis but rather a theoretical model risk estimate. The software used was Maple 2020 (Maplesoft).

## Results

Head and neck cancers had the fastest reported rates of proliferation, with an estimated median (range) doubling time of 99 (61-112) days.^17^ Using this estimated median doubling time, *G* was estimated to be 0.210 months^−1^. Assuming a normal distribution of growth rates, the mean (SD) range was estimated at *G *(2 × σ). Using a geometric mean of estimates based on the lower and upper interquartile range values, σ was estimated to be 0.0282 months^−1^. Colorectal cancers were reported to have intermediate rates of proliferation with an estimated median (range) doubling time of 211 (112-404) days.^18,19^ With this doubling time for colorectal cancers, *G *was estimated to be 0.099 months^−1^, and σ was estimated to be 0.032 months^−1^. Lung cancers were reported to have the slowest rate of proliferation, with a median (interquartile range) doubling time of 348 (222-492) days.^16^ With this doubling time for lung cancers, *G *was estimated to be 0.0598 months^−1^. The interquartile range was estimated by multiplying σ by 0.674, and σ was estimated to be 0.0361 months^−1^.

For head and neck cancer, approximately 60% of new metastases were detected at 14.9 months based on upstaging from stage II to IV during a median (range) treatment delay time of 14.9 (3.6-63.8) months.^17^ This resulted in a *k *of 1.51 × 10^−12^ months^−1^. For colorectal cancer, approximately 17% of new metastases were detected during a median (range) treatment delay time of 5 (2.4-15.7) months.^18,19^ This resulted in a *k *of 1.95 × 10^−12^ months^−1^. For lung cancer, approximately 8% of new metastases were detected at 12 months for the tumors considered in this study (ie, those <1 cm in diameter at *t* = 0).^16^ This resulted in a *k *of 1.40 × 10^−10^ months^−1^.

Model estimates for 2-month and 6-month treatment delay times, based on parameters derived from published literature, are listed in the Table. For head and neck tumors with a 2-month treatment delay, there was an estimated 4.8% (95% CI, 3.4%-6.4%) increase in local tumor control risk and a 0.49% (0.47%-0.51%) increase in new distal metastases risk. A 6-month delay was associated with an estimated 21.3% (13.4-30.4) increase in local tumor control risk and a 6.0% (5.2-6.8) increase in distal metastases risk. For intermediate-growing colorectal tumors, there was a 2.1% (0.7%-3.5%) increase in local tumor control risk and a 2.7% (2.6%-2.8%) increase in distal metastases risk at 2 months and a 7.6% (2.2%-14.2%) increase in local tumor control risk and a 24.7% (21.9%-27.8%) increase in distal metastases risk at 6 months. For slower-growing lung tumors, there was a 1.2% (0.0%-2.8%) increase in local tumor control risk and a 0.19% (0.18%-0.20%) increase in distal metastases risk at 2 months, and a 4.3% (0.0%-10.6%) increase in local tumor control risk and a 1.9% (1.6%-2.2%) increase in distal metastases risk at 6 months. Graphical presentation of the changes in these estimated risks plotted vs delay times up to 12 months is shown in the Figure. For head and neck cancer, mean local TCP at 2 months was 85.2% (2.5th percentile, 83.6%; 97.5th percentile, 86.6%). At 6 months, the mean was 68.7% (2.5th percentile, 59.6%; 97.5th percentile, 76.6%). For probability of new metastases of head and neck cancer at 2 months delay, the mean was 0.49% (2.5th percentile, 0.47; 97.5th percentile, 0.51%). At 6 months delay, the mean was 6.0% (2.5th percentile, 5.23%; 97.5th percentile, 6.8%).

**Table.** Estimated Local TCP Losses and New Metastases Risks for the Analyzed Cancer Types Following 2 and 6 Months of Treatment Delay

Cancer type and delay	% (95% CI)	
	TCP loss	New metastasis risk
Head and neck		
2 mo	4.84 (3.37-6.40)	0.49 (0.47-0.51)
6 mo	21.26 (13.40-30.42)	5.96 (5.23-6.80)
Colorectal		
2 mo	2.06 (0.70-3.54)	2.69 (2.58-2.81)
6 mo	7.57 (2.24-14.22)	24.68 (21.89-27.81)
Lung		
2 mo	1.22 (0.00-2.78)	0.19 (0.18-0.20)
6 mo	4.26 (0.00-10.59)	1.86 (1.60-2.16)

Abbreviation: TCP, tumor control probability.

**Figure.**
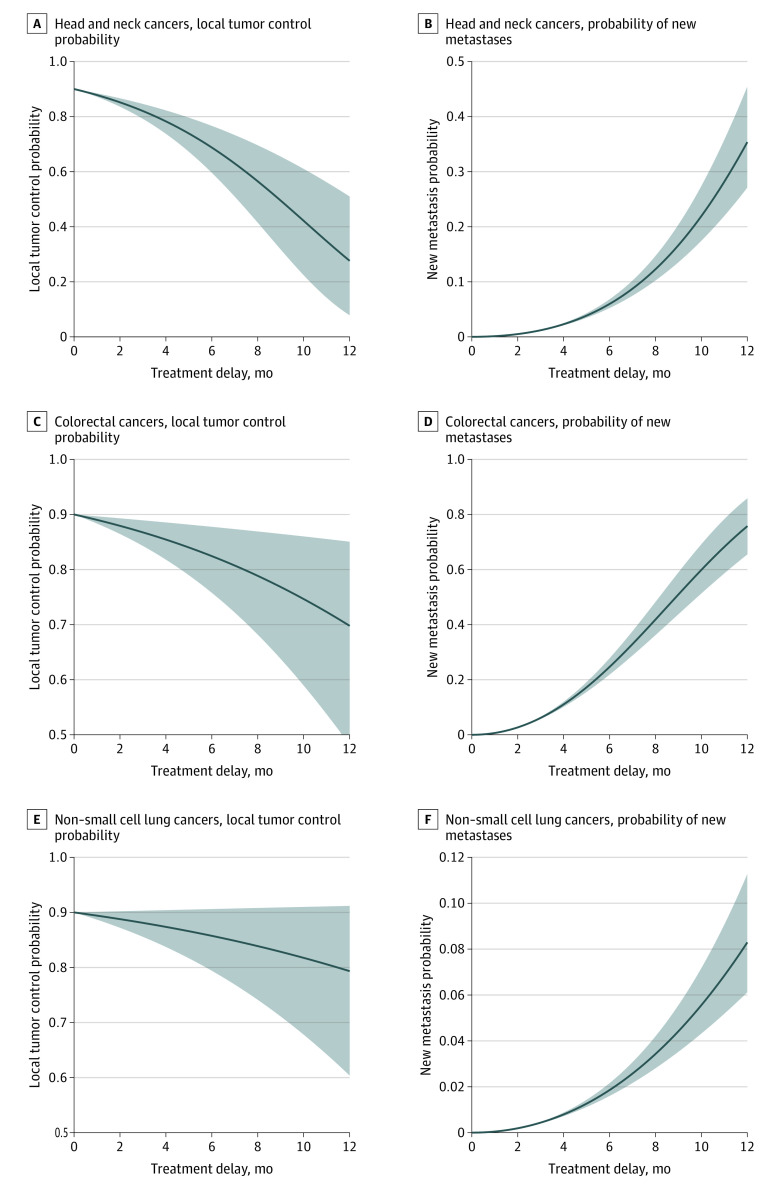
Model Estimations Graphs show data for fast-growing head and neck cancers (A and B), intermediate-growing colorectal cancers (C and D), and slower-growing non–small cell lung cancers (E and F). Solid lines indicate estimates for the mean responses of local tumor control and new metastasis probability to treatment delays; shaded areas indicate the 2.5th and 97.5th percentiles of these estimated responses, so that 95% of patient outcomes are expected to be in between.

## Discussion

Due to the COVID-19 pandemic, there has been renewed interest in understanding the potential clinical impact of treatment delays in cancer treatment. Earlier and recent studies, based mostly on retrospective data or modeling analyses, have noted that delays in cancer treatment by several months may be associated with reductions in treatment effectiveness and increases in mortality risk.^1,3,9,10,11,12,14,15,16,17,18,19,20,21,22,23,24,25,26,27,28,29,30,31,32,33,34,35^ To our knowledge, this is the first study in which the risks of increased metastases and decreased local TCP associated with treatment delays have been quantitatively modeled for cancers with published estimates of tumor doubling times and clinical upstaging rates. The impact of cancer treatment delays due to COVID-19 are already apparent and will likely continue into the foreseeable future.

The quantitative estimates from our study show that, as a proof of principle, delays in cancer treatment by several months are associated with reduced treatment effectiveness. In some patients, these associations can be great, as shown by the range of potential outcomes in the Figure. Rapidly growing tumors, such as head and neck cancers, and rapidly metastasizing tumors, such as colorectal cancers, are likely to have worse estimated outcomes after treatment delay compared with slower growing tumors, such as lung cancers.

Patient population heterogeneity and tumor heterogeneity could lead to a range of potential outcomes from delaying treatment, but the results of this study would estimate a significant loss in locoregional control and a substantial risk of increased incidence of metastatic diagnoses, with significantly higher associated risks with rapidly doubling tumors, such as head and neck malignant neoplasms. Many other cancer types, such as cervical cancers, breast cancers, and anal cancers, have tumor kinetics estimated to lie somewhere along this spectrum of possible outcomes between the head and neck and non–small cell lung cancers modeled here.^22,23,24,29,30,34,35^ The risks associated with treatment delays appear to increase exponentially with any treatment delay interval and become particularly substantial for delays longer than 3 months. The underlying basis behind this exponential association of loss of tumor control and delay interval is due to the fundamental doubling properties of cancer cells and tumors, usually estimated to be on the scale of months. For these reasons, several components of this model change nonlinearly with respect to time, including tumor size, likelihood of tumor control, and the likelihood of new metastasis formation from tumor size.

Our model was based on estimation of tumor kinetics from the clinical literature. In terms of data on tumor kinetics, such as tumor doubling rates and new distal metastases development from treatment delays, the clinical literature is sparse. However, there is significant clinical literature supporting the association between delays in cancer treatment by several months and reductions in treatment effectiveness and increase in mortality risk. A 2020 meta-analysis of 34 studies^9^ on the impact of delay in curative treatment across all 3 major treatment modalities (ie, surgery, systemic treatment, and radiotherapy) included patients with head and neck, colorectal, and lung cancer and showed that even treatment delay intervals as short as 4 weeks were associated with an increase in the risk of death. In that meta-analysis,^9^ the authors noted that the association was even stronger for some radiotherapy and systemic indications, with a 9% and 13% increased risk of death for definitive head and neck radiotherapy and adjuvant systemic treatment for colorectal cancer, respectively. The findings from this meta-analysis are concordant with the findings from our study that the faster-proliferating tumors are associated with greater risks. Another cancer modeling study^23^ estimated a decrease of 8% in local TCP with a 4-week delay in treatment for tumors with a doubling time of 90 days. A systematic review of head and neck cancer management^25^ found an association with treatment delay and worse survival. Other studies have found that time from surgery to the initiation of radiotherapy within 6 weeks was associated with improved recurrence-free or overall survival.^26,27,28^ For patients with cervical cancer, treatment delays greater than 7 weeks from diagnosis of locally advanced disease may be associated with overall survival.^29^ Another study indicated that treatment delays of 4 months or longer for cervical cancer may be associated with a 2-fold or greater increase in the risk of death.^30^ For patients with lung cancer, some studies demonstrate worse survival in patients with delayed diagnosis and treatment, including 2 studies that included patients identified through population-based mass screening.^31,32^ Finally, a 2020 modeling study on the association of COVID-19 with breast and colorectal cancer outcomes estimated a potential 10 000 excess deaths (or 1% increase in deaths) for breast and colorectal cancer during the next decade.^1^

### Limitations

There are limitations and simplifications behind our modeling study. The primary purpose of this study was to establish conceptual estimates to guide clinical decision-making rather than to generate rigorously precise estimates. Tumor growth and metastasis formation are complex biological processes affected by many variables. Numerous modeling strategies for these phenomena, with varying degrees of mechanistic detail, have been proposed and incorporated in the development of our mathematical model.^36,37,38,39^ The situation we considered here—delays in cancer treatment—involves a limited period of a few months, which corresponds to a few tumor doubling times. We believe that during this limited time, a maximally simplified modeling approach with a minimum of parameters is adequate for describing the main patterns of the expected responses to treatment delays in terms of tumor volume and metastasis formation. Consequently, we assumed such a simplified approximation, which consists of exponential tumor growth with normally distributed variability in growth rates between tumors, and a proportional dependence between metastasis hazard and primary tumor cell number. However, more detailed models (eg, Gompertz or power law with metastasis emission proportional to the tumor surface area) would be needed to describe tumor development and metastatic risks over longer periods, such as several years.

In applying our simplified model of cancer treatment delays, we could not adjust for real-world cancer treatment countermeasures implemented in response to the initial COVID-19 risk containment measures. While surgeries, radiation treatments, and chemotherapy treatments are often being delayed, patients with cancer are also receiving alternative strategies, such as selected systemic therapies, as bridging therapeutic strategies. Furthermore, over time, hospitals and clinics have put safety measures in place to allow for safe encounters and to reduce potential treatment delays. Nevertheless, as additional COVID-19 waves continue to affect hospital systems nationally and globally, treatment delays for cancer patients remain relevant.

Third, while the exponential nature of tumor doubling is expected to be the dominant effect in clinical oncology, it is well established that tumor kinetics are also affected by the microenvironment.^40^ It is known that tumors eventually outstrip their blood supply, leading to necrosis and slowing down their exponential growth. Metastatic development in patients occurs through a long, complex series of physiologic processes, including selection and adaptation through invasion, transportation, seeding, and growth into distal sites. Aside from time to treatment, many other variables can influence metastatic progression. The nuances of such complex processes are simplified and approximated in this model. Future clinical studies and more sophisticated models with better estimates of tumor kinetics will allow us to refine and improve the accuracy of these risk estimates, broadening the applicability of quantitative models of risks associated with cancer treatment delays.

## Conclusions

In this study, the detrimental association of treatment delays with cancer treatment effectiveness was greatest for faster-proliferating and faster-metastasizing tumors. With the current COVID-19 pandemic causing disruptions to hospitals and medical care centers nationally and globally, delays in cancer treatments for many patients are inevitable. In addition to challenges with treatment capacities within medical care systems, there are significant barriers, including patient fears of coming to medical centers and potential loss of access to health care because of unemployment. Current guidelines have been implemented based on the rapidity of growth of different malignant neoplasms, tumor stage, possible effects of treatment delays or interruptions, patient-specific considerations, the availability of staff and resources to safely deliver treatment, the potential magnitude of treatment benefit, and the likelihood of a delay impacting outcome.^2,6^ While it may be evident that certain treatment delays will likely be associated with worse cancer outcomes during this present crisis, it is important to develop quantitative models that can help to guide approaches when considering treatment decisions for patients.
